# Adaptation of the Compensatory Stepping Response Following Predictable and Unpredictable Perturbation Training

**DOI:** 10.3389/fnhum.2021.674960

**Published:** 2021-07-15

**Authors:** Naoya Hasegawa, Shintaro Tanaka, Hiroki Mani, Takahiro Inoue, Yun Wang, Kazuhiko Watanabe, Tadayoshi Asaka

**Affiliations:** ^1^Department of Rehabilitation Science, Hokkaido University, Sapporo, Japan; ^2^Graduate School of Health Sciences, Hokkaido University, Sapporo, Japan; ^3^Tianjin Key Laboratory of Exercise Physiology and Sports Medicine, College of Social Sport and Health Science, Tianjin University of Sport, Tianjin, China; ^4^Institute of Sports and Health Science, Hiroshima, Japan

**Keywords:** compensatory stepping, margin of stability, automatic postural response, motor adaptation, postural perturbation

## Abstract

**Background:**

Effective training of the backward step response could be beneficial to improve postural stability and prevent falls. Unpredicted perturbation-based balance training (PBT), widely known as compensatory-step training, may enhance the fear of falling and the patterns of postural muscle co-contraction. Contrastingly, PBT with predictable direction or both direction and timing would suppress the fear and the co-contraction patterns during training, but the efficacy of predictable PBT for unpredictable perturbations is still unknown.

**Objective:**

To compare the adaptation effects of compensatory-step training with and without predictable perturbations on backward stepping against unpredictable perturbations.

**Methods:**

Thirty-three healthy young adults were randomly assigned to one of the following step training groups: Unpredicted, Predicted, and Self-initiated. In training sessions, participants were perturbed to induce a compensatory step with (Predicted group) or without (Unpredicted group) knowledge of the perturbation’s direction or while knowing both the direction and timing of the perturbation (Self-initiated group). In test sessions (pre- and post-training), participants were instructed to recover their postural stability in response to an unpredicted perturbation. The margin of stability (MOS), center of mass (COM) shift, and step characteristics were measured during a backward step in both test and training sessions.

**Results:**

All three groups showed a significant increase in the step length and velocity in the post-training sessions compared to those in the pre-training sessions. Moreover, in the Unpredicted and Predicted groups, but not in the Self-initiated group, the MOS at step contact was significantly increased following the training session. In addition, the Self-initiated group showed a significant increase in COM shift at 50 ms after slip onset during training compared to the Unpredicted and Predicted groups.

**Conclusion:**

Unpredicted and predicted PBT improve step characteristics during backward stepping against unpredictable perturbations. Moreover, the unpredictable PBT and PBT with direction-predictable perturbations enhance the feedback postural control reflected as the postural stability at step contact.

## Introduction

Over 30% of people aged 65 and older experience falls each year, and the frequency of falls increases with age (Kalache et al., 2007). Twenty to thirty percent of falls result in moderate-to-severe injuries, which have a major impact on the quality of life, entail a fear of falling, and further reduce mobility (Sterling et al., 2001). Automatic postural responses to external perturbations, which are critical for fall prevention (Maki and McIlroy, 1997; Mansfield et al., 2015), are impaired in older adults (Horak et al., 1989b; Jensen et al., 2001; Mille et al., 2003).

Older adults implement the stepping strategy in response to smaller perturbations compared to young adults (Jensen et al., 2001; Mille et al., 2003). Backward stepping is a more critical measure than forward stepping because there is lesser distance to the stability margin in the backward direction; therefore, it would have more postural instability and necessitate a faster postural response (Goel et al., 2018). Furthermore, the minimum magnitude of perturbation required to induce a backward step correlated with the future frequency of falls during activities of daily living in older adults (Sturnieks et al., 2013). Moreover, older adults have a shorter step length and lower step velocity than young adults (McIlroy and Maki, 1996; Carty et al., 2011; Kilby et al., 2014; Lee et al., 2014). Considering their inefficient compensatory backward stepping and the importance of compensatory stepping for fall prevention (Carty et al., 2015), effective training of the backward step response is essential to improve postural stability and prevent falls.

Recent reviews have reported that perturbation-based balance training (PBT) improves step velocity, step length, and postural stability following a postural perturbation; however, participants were able to predict the direction of postural perturbation in most studies (Gerards et al., 2017; Okubo et al., 2017). For example, repetitive pulls to the participant’s back (Jobges et al., 2004), repeated tilting of a platform (Barrett et al., 2012), treadmill movement (Patel and Bhatt, 2015), or the release of a cable attached to the participant’s back (Arampatzis et al., 2011) were used for training as well as for assessing the effects of training. Contrastingly, falls often occur following postural perturbations with unpredictable timing and direction, such as slips or trips (Berg et al., 1997). In addition, PBT with unpredictable direction has been reported to increase the length of a backward compensatory step in response to a forward surface perturbation (backward compensatory step) with unpredictable timing and direction (Mansfield et al., 2010). On the other hand, another study showed that PBT with unpredictable perturbations did not increase the length of a backward compensatory step and did not decrease the maximum shift of the body’s center of mass (COM) in response to unpredictable perturbations (Dijkstra et al., 2015).

We also focused on the effect of PBT with perturbation of a platform in a predictable direction and in both predictable direction and timing on a compensatory response under unpredictable conditions in this study. We believe that PBT with direction-predictable and both direction- and timing-predictable perturbations could enhance the central set (Horak and Diener, 1994; Timmann and Horak, 1997; Mochizuki et al., 2008). For example, the repeated direction-predictable perturbation improved the efficiency of the postural response for similar perturbations as reflected in the faster return to an equilibrium position (Horak et al., 1989a). Another study showed that perturbations with both predictable direction and timing induced cortical activities that preceded the onset of the perturbations (Mochizuki et al., 2008). The central set could modify the setting of the central nervous system state in preparation for the execution of the evoked automatic postural responses to postural perturbations by taking into account prior experience with predictable perturbations (Horak et al., 1989a; Jacobs and Horak, 2007; Mochizuki et al., 2008). This, in turn, could influence the compensatory-step response to unpredictable perturbations through feedback mechanisms. Furthermore, PBT with unpredictable perturbations may increase the fear of falling and induce an excessive focus on the motor task compared with PBT with predictable perturbations. This fear of falling and excessive focus on the motor task could attenuate anticipatory postural control (Adkin et al., 2002) and enhance postural muscle co-contraction (Asaka et al., 2008). Contrastingly, PBT with direction-predictable and both direction- and timing-predictable perturbations could decrease the fear of falling and pattern of co-contraction, thereby improving the compensatory-step response to unpredictable perturbations.

Therefore, this study aimed to investigate whether PBT with or without predictable direction or timing improves postural stability during backward stepping against unpredictable perturbations. We hypothesized that PBT with direction-predictable and both direction- and timing-predictable perturbations would improve postural stability during backward stepping against unpredictable perturbations as well as PBT with unpredictable perturbations. The results of this study could help effectively train compensatory-step responses in the field of rehabilitation.

## Materials and Methods

### Participants

Thirty-three healthy young adults with no known neurological or motor disorders participated in this study. They were randomly divided into three groups. The participants were perturbed to induce a compensatory-step response with (Predicted group) or without (Unpredicted group) knowledge of the perturbation’s direction or while knowing both the direction and timing of the perturbation (Self-initiated group). Each participant’s age, gender, height, body weight, and average length of both feet were recorded (Table 1). All participants signed informed consent forms, and the study was approved by the ethics committee of Hokkaido University. The study was conducted in accordance with the Declaration of Helsinki (1964).

**TABLE 1:** Demographic data.

	Unpredicted (*N* = 11)		Predicted (*N* = 11)		Self-initiated (*N* = 11)		*p*-value			
	**Mean**	***SEM***	**Mean**	***SEM***	**Mean**	***SEM***	**All Groups**	**Unp. vs. *Predicted***	**Unp. *vs. Self.***	**Predicted vs. *Self.***
Male/Female	5/6		5/6		5/6		–	–	–	–
Age	23.1	0.4	21.2	0.4	23.1	0.7	***0.010^a^***	***0.010***	*1.000*	*0.094*
Height (cm)	164.8	2.3	162.1	3.3	163.7	2.5	*0.772*	*0.662*	*1.000*	*1.000*
Weight (kg)	58.1	2.7	53.8	2.1	57.1	2.3	*0.433*	*0.053*	*0.075*	*1.000*
Foot length (cm)	23.9	0.4	23.6	0.5	24.3	0.4	*0.463^a^*	*1.000*	*0.585*	*0.272*

*Groups compared using ANOVA or Kruskal–Wallis test and significance level of 0.05 (a: Kruskal–Wallis test). Bold values indicate significant differences between groups at *p* < 0.05. Unp., Unpredicted; Self., Self-initiated; SEM, standard error of mean ($s\,t\,a\,n\,d\,a\,r\,d\,d\,e\,v\,i\,a\,t\,i\,o\,n/\sqrt{t\,h\,e\,n\,u\,m\,b\,e\,r\,o\,f\,p\,a\,r\,t\,i\,c\,i\,p\,a\,n\,t\,s}$).*

### Equipment

Kinematic data were collected using a six-camera three-dimensional motion analysis system (Motion Analysis Corporation, Santa Rosa, CA, United States) at a sampling frequency of 200 Hz. Sixteen reflective markers were attached bilaterally to the following bony landmarks: tip of the mastoid process, acromion process, greater trochanter, lateral femoral condyle, head of fibula, lateral malleolus, head of the second metatarsal, and calcaneus. These markers were used to calculate the COM in the anterior-posterior direction based on the head-arm-trunk model (seven body segments: upper body, thighs, shanks, and feet) (Chang et al., 2017). In addition, a reflective marker was attached on a movable horizontal surface to determine the initiation of the perturbation. The surface contained a platform (74 cm × 74 cm × 10 cm) that could be moved using a controller (7 cm × 5 cm × 3 cm) in the forward or backward directions at a constant velocity of 66 cm/s. Acceleration and deceleration time intervals were approximately 150 ms, and the total duration of perturbation was 500 ms (Asaka et al., 2011). The platform moved 10 cm in each direction. The magnitude of perturbation required to necessitate a backward compensatory-step response was determined based on a previous study (McIlroy and Maki, 1996).

### Procedure

Participants stood on the movable platform with bare feet and arms crossed over their chest and, were instructed to gaze at a point approximately 3 m away. They were also instructed to align their upright posture with a vertical line before each perturbation.

The task consisted of three sessions (pre-training, training, and post-training). In the pre- and post-training sessions, which were intended to trigger a backward step in response to an unpredictable forward surface perturbation, the platform was randomly moved in the forward direction in five trials and in the backward direction in nine trials. The perturbation was randomly produced; it followed a beep sound with a time delay that randomly varied between 1 and 10 s. The participants were instructed to react naturally to the perturbation and avoid falling (Yungher et al., 2012).

The training sessions differed among the groups as follows: (1) Unpredicted: the platform was randomly moved in the forward direction in 30 trials and in the backward direction in 30 trials; (2) Predicted: the platform was moved only in the forward direction in 60 trials. These two groups experienced the perturbations following a beep sound with a random time delay between 1 and 10 s; and (3) Self-initiated: the platform was moved only in the forward direction in 60 trials. In addition, the participants decided when the perturbation occurred; they did this by pushing the button of the controller of the movable platform. Each group underwent 60 trials in the training session, which was divided into four blocks. Participants were instructed to rapidly take a step after the perturbation. They were allowed to observe the surface movement before the pre-training session and rest for 5 min between sessions and between blocks.

### Data Analysis

All signals were processed offline using MATLAB R2018b (The MathWorks Inc., Natick, MA, United States). Data from the motion-capture system were filtered with a 20 Hz low-pass, fourth-order, zero-lag Butterworth filter.

**FIGURE 1:**
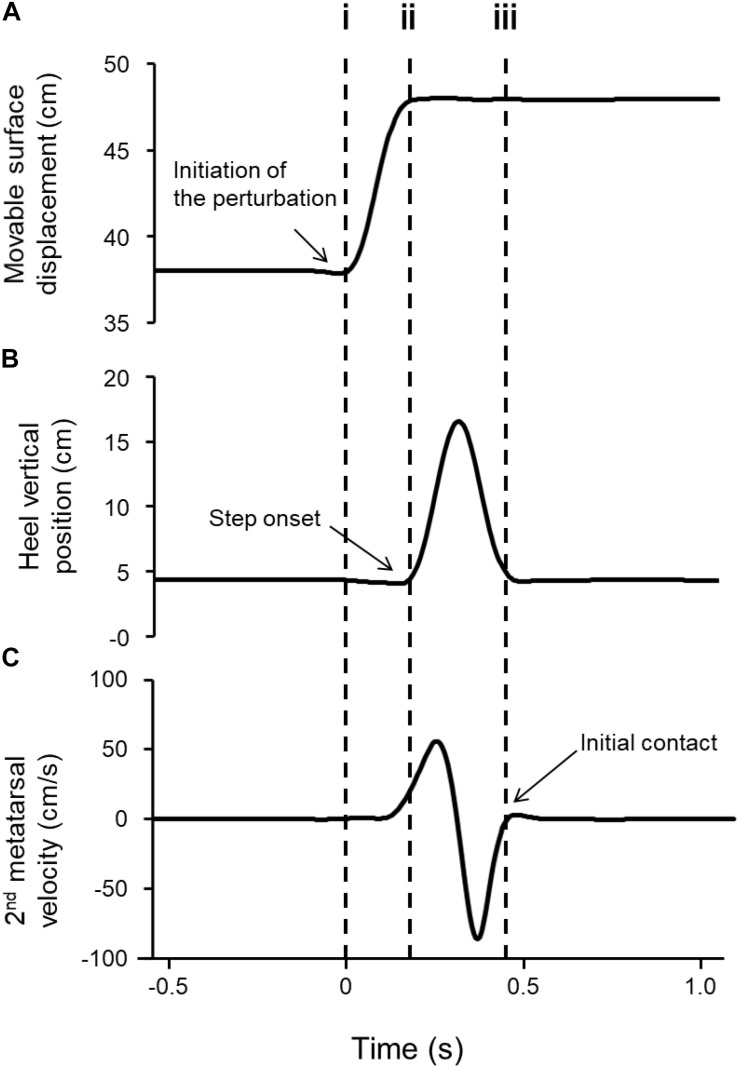
Representative kinematic profiles of the horizontally movable platform and step leg during backward stepping. **(A)** Displacement of the movable platform in the antero-posterior direction, **(B)** heel position in the sagittal plane, and **(C)** vertical velocity of the head of the second metatarsal. The dashed vertical lines indicate (i) movable surface motion onset, (ii) step onset, and (iii) initial contact. Step time is defined as the period between step onset (ii) and initial contact (iii).

Initiation of the perturbation was defined as the moment when the surface started to move in the forward direction (Figure 1A). Initial COM displacement was the displacement between the COM and heel marker at the initiation of the perturbation. Initiation of the step (step onset) was defined as the moment when the vertical heel displacement exceeded two standard deviations of the mean value (Figure 1B). The mean value was calculated during 1 s before the initiation of the perturbation. Termination of the step (initial contact) was defined as the moment when the vertical velocity of the head of the second metatarsal exceeded zero after its deceleration phase (Figure 1C). Step time was defined as the interval from step onset to initial contact. Step length was defined as the difference between the displacement of the head of the second metatarsal at step onset and initial contact. Step velocity was calculated as step length divided by step time.

**FIGURE 2:**
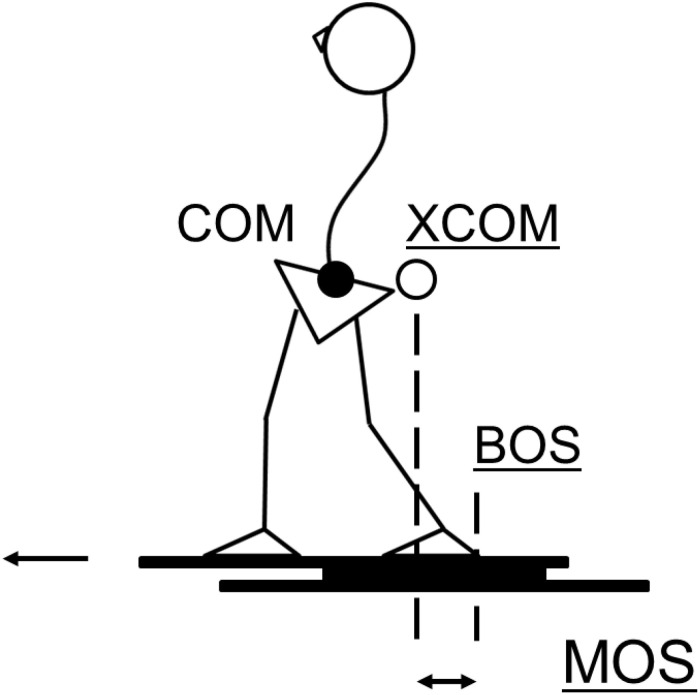
Schematic diagram showing the parameters used to compute the margin of stability at backward initial contact in the antero-posterior direction. The MOS is calculated as follows: MOS = edge of BOS – XCOM, i.e., the difference between the two dashed lines. Edge of BOS is represented by the heel marker on the step leg. XCOM is computed using the formula (XCOM = dCOM + vCOM/$\sqrt{g/l}$), where *dCOM* is the COM displacement, *vCOM* is the COM velocity, *g* is the acceleration of gravity, and *l* is the distance of the COM from the axis of the ankle joint in the sagittal plane. MOS, margin of stability; XCOM, extrapolated center of mass; BOS, base of support; COM, center of mass.

In addition, the margin of stability (MOS) was calculated to evaluate postural stability as the distance from the edge of the base of support to the extrapolated COM (XCOM) in the sagittal plane (Figure 2). XCOM was calculated using the followed formula (Hof et al., 2005):

(1) $$d\,C\,O\,M+v\,C\,O\,M/\sqrt{g/l}$$

where dCOM is the COM displacement, vCOM is the COM velocity, g is the acceleration of gravity, and l is the distance from the ankle joint axis (the lateral malleolus marker) to the COM displacement in the sagittal plane. MOS was calculated immediately before (MOS onset) and after (MOS contact) the step response. Since backward steps in response to forward perturbations were analyzed, the heel marker was considered as the edge of the base of support. Thus, a positive MOS indicates that the XCOM is within the base of support, resulting in a stable body configuration, while a negative MOS indicates an unstable body configuration and a need to take additional steps to avoid a fall.

It was assumed that self-initiated trials trained feedforward control because participants were able to decide the direction and time of the perturbation. Therefore, COM dynamics immediately before the postural response were investigated in the training session. COM displacement was defined as the displacement of the COM between 0 and 50 ms (COM displacement^50ms^). COM velocity was calculated as the COM velocity at 50 ms after the perturbation (COM velocity^50ms^). Positive values of COM displacement^50ms^ and COM velocity^50ms^ indicate that the COM shifted in the forward direction with respect to the COM at the initiation of perturbation, and vice versa. Since the latency of a spinal reflex, which is the shortest reflex in feedback control, is 50 ms (Hwang et al., 2014), COM displacement^50ms^ and COM velocity^50ms^ are influenced by feedforward control.

The backward step in response to forward perturbation was analyzed in both the test and training sessions. To determine whether adaptation occurs due to the forward perturbations experienced in the pre-training session, we calculated the maximum COM shift and MOS in the forward direction. Since a few compensatory forward steps in response to unpredictable perturbations were observed in the pre-training session (<30% of all trials in each participant), the trials without a forward step in response to backward platform movement were analyzed in the test session. The maximum COM shift and MOS in the forward direction were calculated as the difference between the maximum value after the initiation of the perturbation and mean value during 1 s before the initiation of the perturbation. The initial COM displacement, step length, MOS onset, MOS contact, COM displacement^50ms^, and the maximum COM shift and MOS in the forward direction were divided by foot length to ensure a normal distribution.

### Statistical Analysis

The distribution of the demographic data of the three groups (Unpredicted, Predicted, and Self-initiated) was determined using the Shapiro-Wilk test. One-way analysis of variance was used for between-group comparisons of height and weight. Since the data were not normally distributed, the Kruskal–Wallis test was used to compare the age and average length of both feet between the three groups. Bonferroni pairwise comparison and Dunn-Bonferroni comparison were used for *post hoc* analysis of variables with and without normal distributions, respectively.

Two-way mixed-design analysis of variance was used with the factors *Test session* (pre- and post-training) and *Group* (Unpredicted, Predicted, and Self-initiated) to analyze possible differences in all the above-mentioned outcome measures except for COM displacement^50ms^ and COM velocity^50ms^. In addition, to determine the immediate adaptation effects of each training method, the delta of each outcome measure, including COM displacement^50ms^ and COM velocity^50ms^, was compared among the three groups using a one-way analysis of variance. The delta values were calculated by subtracting the mean pre-training value from the value of the last five backward steps in the training session. *Post hoc* analysis was performed using the Bonferroni pairwise comparison.

To determine the step variables related to the MOS, relationships between the MOS and stepping outcomes (step onset, step time, step length, and step velocity) at the pre-training in all three groups were calculated using Pearson’s correlation coefficient. The statistical analyses for the outcome measures and correlations were conducted using SPSS Statistics version 25.0 (IBM, Armonk, NY, United States). The level of significance was set at *p* < 0.05.

## Results

The three groups did not differ with respect to height, weight, and foot length (Table 1). However, the Predicted group was significantly younger than the Unpredicted group (*p* = 0.010), but not more than the Self-initiated group (*p* = 0.094).

**FIGURE 3:**
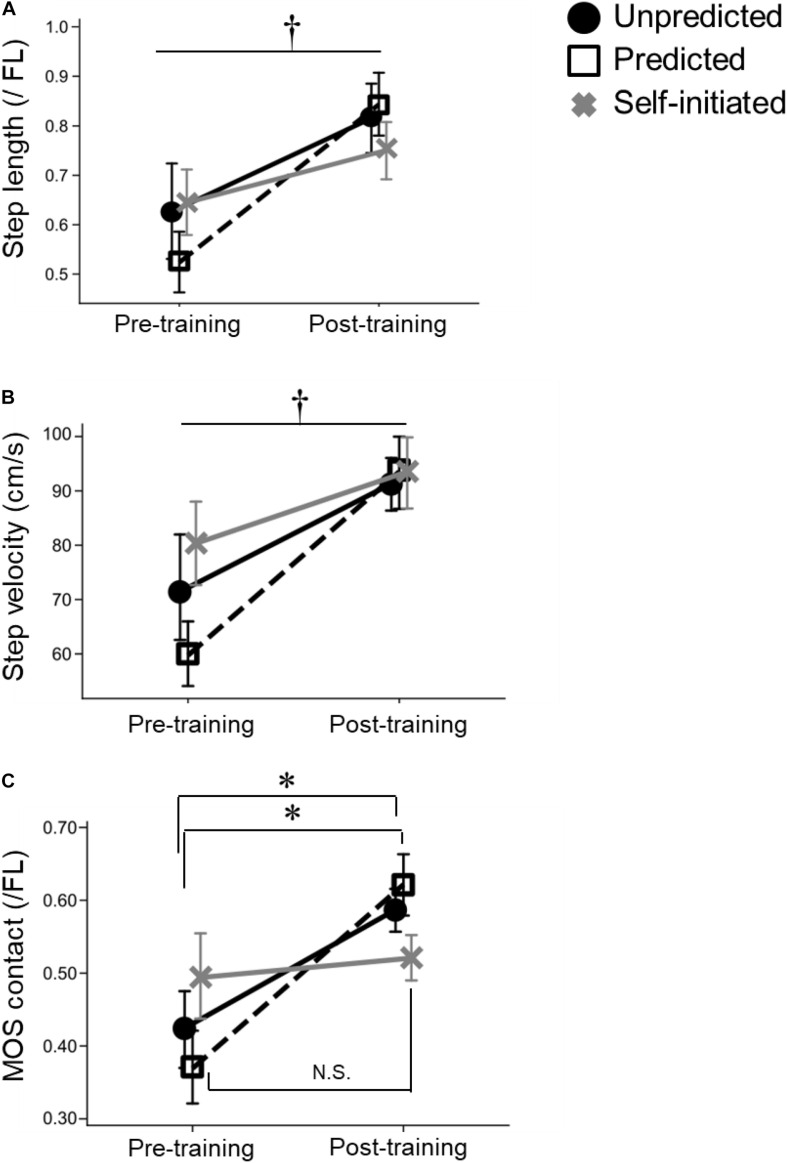
Significant effects of Unpredicted and Predicted step training on stepping outcomes and margin of stability. Mean and standard error of mean plots of two stepping outcomes **(A,B)** and one MOS outcome **(C)**: **(A)** Step length, **(B)** Step velocity, and **(C)** MOS at initial contact. Black circles represent the Unpredicted step training group, white squares represent the Predicted step training group, and gray crosses represent the Self-initiated step training group. Error bars show the SEM, (*) indicate *p*-values less than 0.05 on *post hoc* analysis, and the (†) indicates a significant main effect of the Test session. MOS, margin of stability; SEM, standard error of mean ($s\,t\,a\,n\,d\,a\,r\,d\,d\,e\,v\,i\,a\,t\,i\,o\,n/\sqrt{t\,h\,e\,n\,u\,m\,b\,e\,r\,o\,f\,p\,a\,r\,t\,i\,c\,i\,p\,a\,n\,t\,s}$); N.S., non-significance; FL, foot length.

No significant main effects or interactions were found between *Test session* and *Group* for COM displacement immediately before the perturbation, i.e., initial COM displacement (Table 2). This indicated that no type of step training changed the posture at rest before the unpredictable perturbation at post-training. Contrastingly, step training was found to have a significant main effect of *Test session* on step parameters during backward stepping (step length: *F*_1,30_ = 23.556, *p* < 0.001; step velocity: *F*_1,30_ = 32.754, *p* < 0.001). Specifically, step length and step velocity were significantly increased after step training in all three groups (Figures 3A,B). No significant main effects of *Group* or interactions were found between *Test session* and *Group* for these outcomes, indicating that the three types of step training exhibited similar effects.

**TABLE 2:** Results from the two-way mixed-design analysis of variance for each step measure.

	Baseline, Mean (SEM)			Chance after each training, Mean (SEM)					
Outcomes	Unpredicted	*Predicted*	Self-initiated	Unpredicted	*Predicted*	Self-initiated	Fixed factor	*F* value	*p* Value
COM initial	0.47	0.43	0.46	0.00	−0.02	−0.02	Test	1.995	*0.168*
(/FL)	(0.01)	(0.02)	(0.02)	(0.02)	(0.01)	(0.02)	Group	1.982	*0.155*
							Interaction	0.769	*0.472*
Step onset	0.32	0.26	0.35	−0.06	0.01	−0.05	Test	2.190	*0.149*
(s)	(0.03)	(0.03)	(0.03)	(0.03)	(0.05)	(0.03)	Group	1.777	*0.186*
							Interaction	1.096	*0.347*
Step time	0.20	0.20	0.19	0.00	0.01	0.00	Test	0.312	*0.580*
(s)	(0.01)	(0.01)	(0.01)	(0.02)	(0.01)	(0.01)	Group	0.864	*0.432*
							Interaction	0.066	*0.937*
**Step length**	**0.62**	**0.52**	**0.64**	**0.19**	**0.32**	**0.12**	**Test**	**23.556**	***<0.001***
**(/FL)**	**(0.10)**	**(0.07)**	**(0.07)**	**(0.08)**	**(0.08)**	**(0.06)**	Group	0.089	*0.915*
							Interaction	2.168	*0.132*
**Step velocity**	**71.5**	**59.7**	**80.3**	**19.9**	**33.8**	**13.2**	**Test**	**32.754**	***0.001***
**(cm/s)**	**(9.95)**	**(6.27)**	**(7.69)**	**(8.29)**	**(7.03)**	**(4.31)**	Group	0.656	*0.526*
							Interaction	2.433	*0.105*
MOS onset	−0.04	−0.05	−0.05	0.05	0.01	−0.04	Test	0.129	*0.722*
(/FL)	(0.04)	(0.04)	(0.04)	(0.05)	(0.03)	(0.02)	Group	0.877	*0.427*
							Interaction	1.532	*0.233*
**MOS contact**	**0.42**	**0.37**	**0.49**	**0.16**	**0.25**	**0.03**	**Test**	**27.311**	***0.001***
**(/FL)**	**(0.05)**	**(0.05)**	**(0.06)**	**(0.05)**	**(0.04)**	**(0.05)**	Group	0.025	*0.976*
							**Interaction**	**5.424**	***0.010***

*Values at baseline (pre-training) and changes after each training (differences between pre- and post-training) are presented. Bold values indicate significant effects at *p* < 0.05. COM, center of mass; FL, foot length; MOS, margin of stability; SEM, standard error of mean ($s\,t\,a\,n\,d\,a\,r\,d\,d\,e\,v\,i\,a\,t\,i\,o\,n/\sqrt{t\,h\,e\,n\,u\,m\,b\,e\,r\,o\,f\,p\,a\,r\,t\,i\,c\,i\,p\,a\,n\,t\,s}$).*

A significant interaction between *Test session* and *Group* was found for the MOS at initial contact (*F*_2,30_ = 5.424, *p* = 0.010). Specifically, *post hoc* analysis revealed that the Unpredicted and Predicted groups had a significantly increased MOS at initial contact following training (*p* = 0.010 and *p* = 0.035, respectively), but the MOS of the Self-initiated group did not change (*p* = 0.621; Table 2 and Figure 3C), indicating that both Unpredicted and Predicted step training improved postural stability during backward stepping.

**FIGURE 4:**
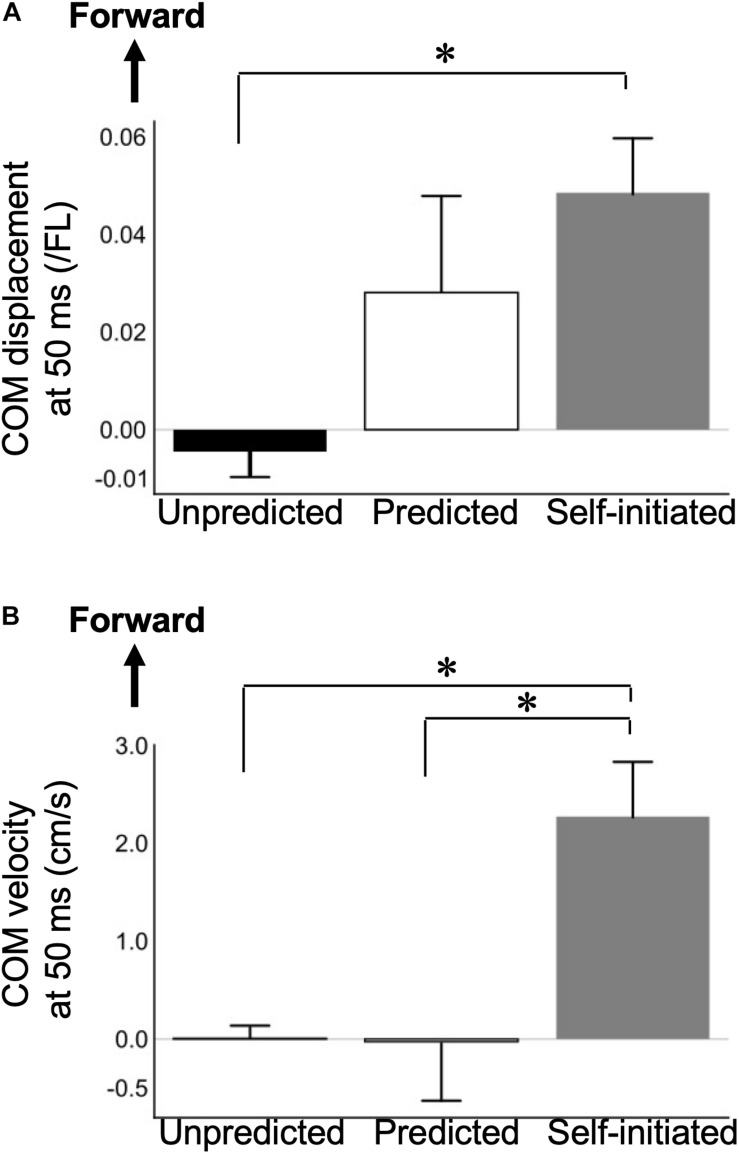
Significant differences in the effect of step training on center of mass outcomes in the Self-initiated step training group compared to the Unpredicted and Predicted step training groups. Mean and standard error of mean plots of two COM outcomes: **(A)** COM displacement and **(B)** COM velocity at 50 ms after the perturbation. Bar plots summarize the change in each measure before versus during step training. Error bars show the standard error of mean. Positive values indicate that the COM shifted in the forward direction with respect to the COM at the initiation of perturbation, and vice versa. *p < 0.05; COM, center of mass; FL, foot length.

The Unpredicted, Predicted, and Self-initiated groups significantly differed in the change of COM displacement and velocity between 0 and 50 ms (*F*_2,30_ = 3.889, *p* = 0.032 and *F*_2,30_ = 7.375, *p* = 0.002, respectively; Table 3). *Post hoc* analysis revealed a significantly greater increase in the COM displacement at 50 ms following Self-initiated step training than that following Unpredicted step training (*p* = 0.029; Figure 4A). The COM velocity at 50 ms following Self-initiated step training was also significantly increased compared to those following Unpredicted and Predicted step trainings (*p* = 0.007 and *p* = 0.007, respectively; Figure 4B).

**TABLE 3:** Results from the one-way analysis of variance for each change of outcome measure between the pre-training and training sessions.

	Unpredicted		Predicted		Self-initiated		*p*-value			
	Mean	*SEM*	Mean	*SEM*	Mean	*SEM*	All Groups	Unp. vs. *Predicted*	Unp. vs. *Self.*	Predicted vs. *Self.*
**COM displacement^50ms^ (/FL)**	−**0.004**	**0.006**	**0.028**	**0.019**	**0.049**	**0.012**	**0.032**	0.229	**0.029**	0.884
**COM velocity^50ms^ (cm/s)**	**0.01**	**0.14**	−**0.03**	**0.63**	**2.27**	**0.53**	***0.002***	*1.000*	***0.007***	***0.007***
Step onset (s)	−0.07	0.03	−0.05	0.03	−0.11	0.04	*0.341*	*1.000*	*1.000*	*0.452*
Step time (s)	0.01	0.02	0.02	0.01	0.04	0.03	*0.496*	*1.000*	*0.770*	*1.000*
Step length (/FL)	0.24	0.06	0.35	0.10	0.24	0.11	*0.613*	*1.000*	*1.000*	*1.000*
Step velocity (cm/s)	22.9	7.5	33.1	9.1	12.1	5.3	*0.159*	*1.000*	*0.948*	*0.172*
MOS onset (/FL)	0.07	0.05	0.11	0.04	0.14	0.04	*0.482*	*1.000*	*0.695*	*1.000*
MOS contact (/FL)	0.21	0.04	0.31	0.06	0.19	0.08	*0.340*	*0.719*	*1.000*	*0.539*

*Change values between pre-training and the last 5 trials of training session are presented. Bold values indicate significant differences between groups at *p* < 0.05. COM, center of mass; COM displacement^50ms^, COM displacement between 0 and 50 ms; COM velocity^50ms^, COM velocity at the 50 ms after perturbation; FL, foot length; MOS, margin of stability; Unp., Unpredicted; Self., Self-initiated; SEM, standard error of mean ($s\,t\,a\,n\,d\,a\,r\,d\,d\,e\,v\,i\,a\,t\,i\,o\,n/\sqrt{t\,h\,e\,n\,u\,m\,b\,e\,r\,o\,f\,p\,a\,r\,t\,i\,c\,i\,p\,a\,n\,t}$).*

Few participants took a compensatory forward step in response to backward platform movement in both the pre-training (24.2 ± 30.3% of all trials) and post-training sessions (37.0 ± 40.0% of all trials). No significant main effects of *Test session* (*p* = 0.059 and *p* = 0.274, respectively) and *Group* (*p* = 0.130 and *p* = 0.355, respectively) or interactions between *Test session* and *Group* (*p* = 0.939 and *p* = 0.494, respectively) were found for the maximum COM shift and MOS in the forward direction, indicating that none of the three types of step training exhibited training effects for unpredictable backward platform movements.

Pearson’s correlation analysis revealed a significant positive association of MOS contact with step length (*r* = 0.73, *p* < 0.001) and step velocity (*r* = 0.73, *p* < 0.001) (Table 4). In addition, MOS at step onset was significantly negatively associated with step onset (*r* = −0.43, *p* = 0.021) and step velocity (*r* = −0.43, *p* = 0.019).

**TABLE 4:** Correlation of MOS measures with step measures.

	MOS onset (/FL)		MOS contact (/FL)	
	*r* value	*p*-value	*r* value	*p*-value
Step onset (s)	−**0.43**	***0.021***	0.15	*0.485*
Step time (s)	0.10	*0.652*	0.34	*0.083*
Step length (/FL)	−0.31	*0.115*	**0.73**	***0.001***
Step velocity (cm/s)	−**0.43**	***0.019***	**0.73**	***0.001***

*R-values and p-values are presented. Bold values indicate significant effects at p < 0.05. MOS, margin of stability; FL, foot length.*

## Discussion

This study investigated the adaptation effects of PBT under predictable or unpredictable conditions on a backward step response against an unpredictable perturbation. All three types of PBT enhanced the characteristics of the backward step response to an unpredictable perturbation, even when the participants predicted the timing and direction of the perturbation during the step training. In addition, the MOS at step contact was increased by PBT with unpredictable or direction-predictable perturbations, and the increase in the MOS was correlated with a longer step length and faster step velocity. Our findings firstly revealed that PBT against a perturbation of predictable direction improved the postural stability at backward step contact against an unpredictable perturbation.

Consistent with previous studies on PBT with unpredictable perturbations, our findings demonstrated that PBT with unpredictable perturbations increased the length and velocity of the backward compensatory step in response to an unpredictable perturbation (Patel and Bhatt, 2015). Moreover, we found that these effects were induced regardless of whether the perturbations used in the training were predictable. It is well known that the adaptive response to external postural perturbation is largely influenced by the central set developed from prior experience (Timmann and Horak, 1997; Bolton, 2015). Therefore, during the first exposure to external perturbations, the central nervous system would employ a feedback system in order to execute a compensatory step to maintain equilibrium (Bhatt et al., 2006). In contrast, with repeated exposure to external postural perturbations, postural responses might be modified based on prior experiences (Barrett et al., 2012). These modified stepping responses are fast preconditioned responses that bypass some stages of information processing and can be executed in response to similar stimuli (Schmidt and Lee, 2011). Interestingly, previous studies showed that this modified response did not involve any changes in the background or onset of muscle activity (Welch and Ting, 2014; Dijkstra et al., 2015). Thus, it is faster not because it pre-determines or prepares motor activities, but because it references or recalibrates a previously constructed motor memory without the need of developing a new motor pattern. Therefore, PBT may lead to the formation of motor memories that could make the central nervous system trigger a rapidly modified response to attain the optimal level of stability even when the external postural perturbation is unpredictable, regardless of whether the PBT used predictable or unpredictable perturbations. Step training with predictable perturbations might be useful in individuals with postural instability as it is associated with lower fear of falling and patterns of postural muscle co-contraction than training with unpredictable perturbations (Adkin et al., 2002; Asaka et al., 2008).

In line with our hypothesis, PBT with unpredictable perturbations and perturbations of a predictable direction induced greater dynamic postural stability than the pre-training test, which was shown by an increased MOS at step contact. Furthermore, a longer step length and faster step velocity led to greater postural stability immediately after compensatory backward stepping. These findings may indicate that unpredictable and direction-predictable PBT increase dynamic postural stability in response to external postural perturbations by a mechanism similar to the one making the compensatory backward step faster and longer. In contrast, PBT with direction- and timing-predictable perturbations showed no significant change in the MOS at step contact in response to unpredictable perturbations compared to pre-training trials. These differences may be explained based on the two components of the protective step strategy: proactive (i.e., pre-slip) and reactive (i.e., post-slip). Reactive components, such as increased step length and step velocity, could contribute to enhanced stability by providing a greater stabilizing moment through the contact force to decelerate the COM excursion (Maki and McIlroy, 1997). On the other hand, previous studies have reported proactive adaptations while performing more dynamic postural tasks, such as sit to stand tasks and walking (Bhatt et al., 2006; Pai et al., 2010; Parijat and Lockhart, 2012); participants changed their posture in anticipation of perturbations. Thus, proactive components against slips are characterized by an anterior shift of the COM and increased COM velocity before slip onset to reduce the excursion of the XCOM. However, we observed no change in COM states before (Table 2), but not immediately after (Figure 4), slip-onset during PBT with both direction- and timing-predictable perturbations. Therefore, timing-predictable perturbations induced more dependence on proactive components to maintain postural stability and may be inadequate to strengthen the reactive components that contribute to the response to unpredictable perturbations.

This study has several limitations. First, this study included a small number of young participants, which may overlook differential effects between predictable and unpredictable perturbation training. Future studies with a larger sample size and on older adults with a fear of falling or individuals with balance disorders should be conducted. Second, the experimental design should include follow-up tests to assess the motor learning following training. Third, the results at post-training may have been affected because adaptations to repeated postural perturbations occur relatively quickly after being delivered during the pre-training session, and motor adaptations could have been elicited in all three training groups. Fourth, we cannot investigate the transfer effects for forward step responses to unpredictable perturbations since the magnitude of the perturbations was too small to induce a forward compensatory step (<30% of all trials during pre-training in each participant). In the Unpredicted group, the presence of transfer effects for forward step responses in the training session could have contributed to the enhancement of the backward step response in the post-training session. Fifth, we did not evaluate foot loading when the participants initially stood on the movable surface. This parameter could have affected the initial step response (Mancini et al., 2009). Finally, the change in neuroimaging findings, muscle activities, and the fear of falling should be investigated to understand and confirm adaptation to external postural perturbations.

This study showed that compensatory-backward-step training with direction-predictable or unpredictable perturbations resulted in similar improvements in step characteristics and postural stability at step contact in response to unpredictable perturbations. In contrast, PBT with direction- and timing-predictable perturbations improved anticipatory postural responses preceding compensatory step responses, but no improvement was observed in the postural stability at step contact in response to an unpredictable perturbation. The results of this randomized trial provide fundamental evidence for the use of PBT with direction-predictable perturbations as well as unpredictable perturbations to enhance step characteristics and postural stability at step contact.

## Data Availability Statement

The raw data supporting the conclusions of this article will be made available by the authors, without undue reservation.

## Ethics Statement

The studies involving human participants were reviewed and approved by Hokkaido University Ethics Committee. The participants provided their written informed consent to participate in this study.

## Author Contributions

NH: conception, investigation, and methodology of the study, data analysis, and drafting and revising the manuscript. ST and HM: conception, investigation, and methodology of the study, data analysis, and editing the manuscript. TI: data analysis and revising the manuscript. YW and KW: methodology of the study and editing the manuscript. TA: conception and supervision of the study and editing and revising the manuscript. All authors contributed to the article and approved the submitted version.

## Conflict of Interest

The authors declare that the research was conducted in the absence of any commercial or financial relationships that could be construed as a potential conflict of interest.
